# Safe and Effective Treatment Protocol for Facial Pigmentary Disorders in Asian Patients: A Key to Success for Plastic Surgery Residents With Limited Clinical Experience in Aesthetic Dermatology

**DOI:** 10.7759/cureus.83467

**Published:** 2025-05-04

**Authors:** Daiki Kitano, Misako Morita, Chikara Takekawa

**Affiliations:** 1 Plastic Surgery, Kobe University, Kobe, JPN; 2 Plastic and Aesthetic Surgery, Koyama Clinic, Kobe, JPN; 3 Plastic and Aesthetic Surgery, Liki Clinic Kobe, Kobe, JPN

**Keywords:** chemical bleaching, laser, melasma, resident clinic, senile lentigo

## Abstract

Introduction: Asian patients seeking facial rejuvenation treatments have a high incidence of melasma and senile lentigo, collectively known as aging complex pigmentation (ACP), making accurate diagnosis essential for appropriate treatment planning. This study evaluates the versatility of a treatment protocol established by attending plastic surgeons for residents with limited clinical experience in aesthetic dermatology.

Methods: This retrospective observational study included 27 consecutive Asian patients who visited our clinic between December 2020 and December 2021 with a chief complaint of facial pigmentary disorders. Patients suspected of having ACP underwent protocol-based treatment, which consisted of oral medications (tranexamic acid, vitamin C, and vitamin E), chemical bleaching (topical hydroquinone and retinoic acid ointment), and laser therapy. Treatment outcomes were assessed in a blinded manner by two board-certified attending physicians using the Melasma Area and Severity Index (MASI).

Results: All patients were diagnosed and treated exclusively by a resident. Six patients with ACP completed the combination therapy without any major adverse effects. The mean MASI score showed a significant improvement, decreasing from 19 before treatment to 2.8 after treatment (p=0.0054; unpaired t-test).

Discussion: The combination therapy provided satisfactory outcomes, regardless of the resident's clinical experience. Residents acquire essential hands-on experience in aesthetic dermatology while minimizing the risk of adverse outcomes. Moreover, the standardized treatment protocol streamlined the training process, reducing the time and effort needed from attending surgeons, ultimately improving team efficiency.

Conclusion: Our findings suggest that this standardized treatment protocol is an effective and safe approach for managing ACP while providing residents with valuable clinical training in aesthetic dermatology. Implementing such structured protocols in academic settings may enhance both patient outcomes and the educational experience of trainees.

## Introduction

Facial pigmentary disorders are among the most common concerns for patients seeking treatment from aesthetic dermatologists. Asian patients have a high incidence of melasma and senile lentigo (SL) [1], collectively known as aging complex pigmentation (ACP) [2]. Q-switched laser is a conventional treatment modality for SL; however, it is ineffective against melasma and can even exacerbate pigmentation in patients with melasma [3]. Therefore, accurately distinguishing melasma from other pigmentary disorders is essential for the proper treatment of patients with ACP.

However, this requires substantial clinical experience, making sole practice by residents unrealistic for on-the-job training. Additionally, the difficulty of obtaining patient cooperation for resident training further complicates resident involvement in aesthetic dermatology. From the attending surgeons' perspective, the desire to avoid unfavorable outcomes that could damage the patient-doctor relationship often leads to hesitancy in providing clinical opportunities to residents. As a result, only a few residents gain hands-on experience in this field.

We have previously reported a safe and effective treatment protocol for pigmentary disorders in Asian patients [4]. The protocol combines oral medications (tranexamic acid (TA), vitamin C, and vitamin E), chemical bleaching (topical hydroquinone (HQ) and retinoic acid (RA) ointment), and laser treatment, offering a comprehensive solution for the primary complaints of ACP patients. Furthermore, the protocol strictly defines the treatment modalities and the appropriate timing for their application, reducing intra-operator variance and ensuring promising clinical outcomes. Given that the protocol provides satisfactory results regardless of the physician's clinical experience, it serves as an optimal tool for the on-the-job training of residents in aesthetic dermatology. This study aims to validate the protocol's versatility in real clinical settings, where a plastic surgery resident carried out sole practice at an aesthetic dermatology clinic.

## Materials and methods

Patients

A fourth-year plastic surgery resident (DK) was assigned to conduct weekly initial outpatient consultations at Koyama Clinic, a private aesthetic dermatology practice in Kobe, Japan. The clinic operated on a single-doctor basis, with the resident handling patient care primarily through sole practice, seeking assistance from the on-call attending doctor only in cases of emergencies. The subjects of this study were outpatients with a chief complaint of pigmentary disorders who visited the clinic between January and December 2021. Combination therapy [4], consisting of oral TA and vitamins, HQ and RA ointment, as well as laser treatment, was indicated for patients suspected to have ACP. Patients who either did not consent to the combination therapy or were not suspected to have ACP were excluded from this study. A retrospective cohort analysis was conducted based on medical records, considering the patients' age, sex, clinical diagnosis, treatment, and outcome. Written informed consent for publication, including the use of facial images, was obtained from the patients.

Treatment protocol

**Figure 1:**
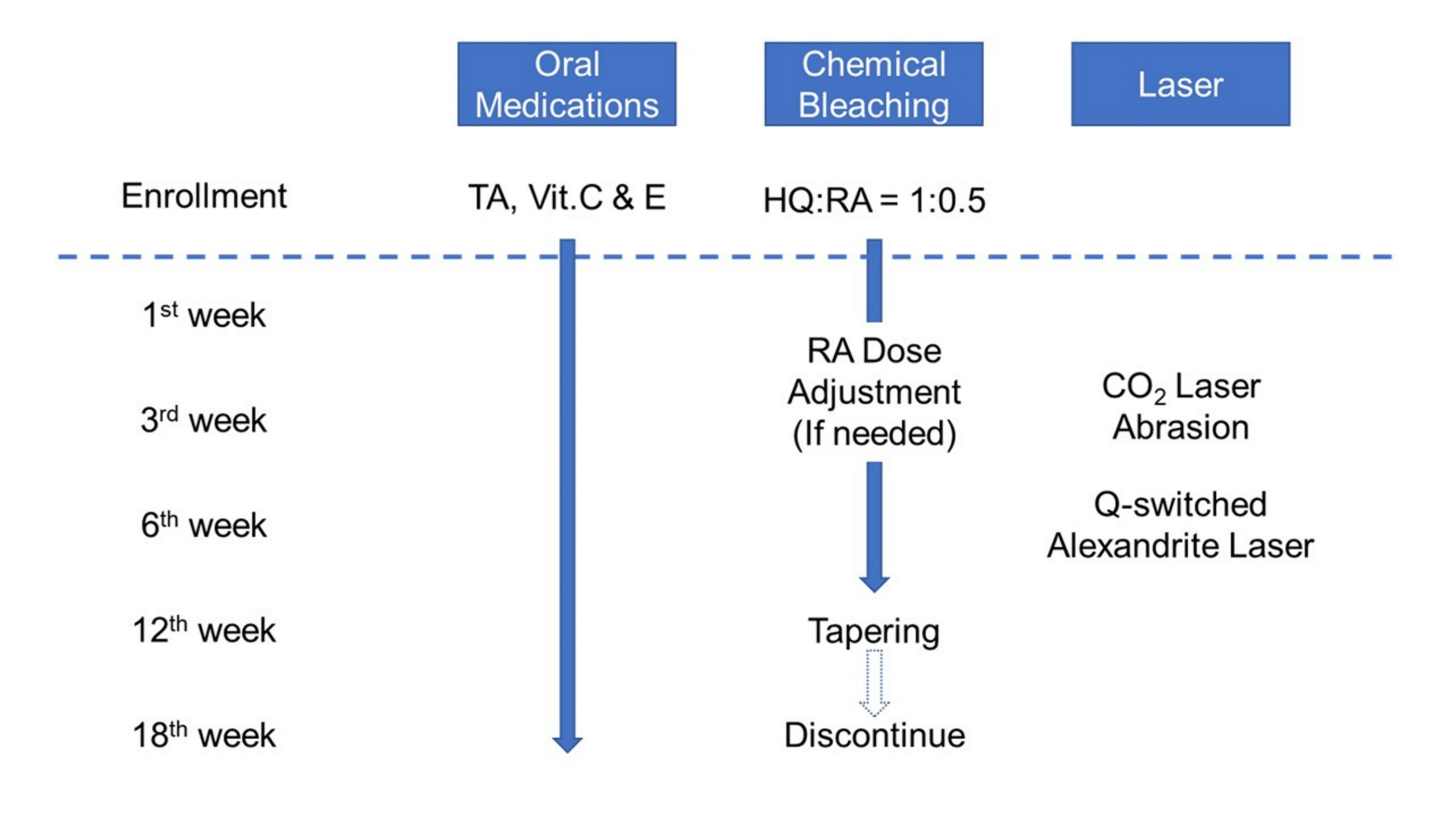
The protocol of our combination therapy Oral medications, chemical bleaching, and laser therapy were combined to effectively treat a broad spectrum of common facial pigmentary disorders typically encountered in clinical practice. By prioritizing oral medications and chemical bleaching, melasma can be effectively controlled by the sixth week, before proceeding with Q-switched alexandrite laser treatment. CO2: carbon dioxide; HQ: hydroquinone; RA: retinoic acid; TA: tranexamic acid; Vit.: vitamin

In accordance with the treatment protocol established by attending surgeons [4] (Figure 1), patients suspected of having ACP were prescribed oral TA (Tranexamic Acid Tablets®, Nichiiko, Tokyo, Japan) 1,000 mg/day, vitamin C (CINAL® Combination Tablets, Shionogi, Tokyo, Japan) 1,000 mg/day, and vitamin E (Juvela® Tablets, Alfresa Pharma, Osaka, Japan) 50 mg/day, along with daily chemical bleaching using ZO® (ZO Skin Health Inc., Irvine, California, United States). Patients were instructed to apply Milamin® in the morning and Miramix® in the evening for the first week, both of which are HQ-based formulations. After confirming no allergic reactions to HQ, patients were then allowed to start using 0.1% RA ointment (Obagi® Tretinoin 0.1% Cream, Obagi Cosmeceuticals LLC, Long Beach, California, United States). We adopted a "1:0.5 policy" for the mixture: 1 push of Miramix® was mixed with half the amount of 0.1% RA. The amount of 0.1% RA was adjusted based on the degree of exfoliation and erythema observed.

In the third week after the initiation of daily chemical bleaching, CO2 laser abrasion (UltraPulse® Encore, Lumenis Be Japan Co., Ltd., Tokyo, Japan) was performed for seborrheic keratosis (SK) and nevocellular nevus (NCN). The treated area was covered with Duoactive ET® (ConvaTec Japan K.K., Tokyo, Japan), a hydrocolloid occlusive dressing, for two weeks, allowing patients to continue daily chemical bleaching except for the abraded area. In the sixth week, Q-switched laser (Alex® Laser, Syneron Candela, Marlborough, Massachusetts, United States), with a fluence range of 7-8 J/cm², was applied for treatment of SL and freckles. The irradiated area was covered with 3M™ Micropore™ Tan Surgical Tape (3M Japan Limited, Tokyo, Japan) for two weeks. Patients were instructed to continue daily chemical bleaching on their face, avoiding the area of laser irradiation. The RA dosage was gradually reduced in the 12th week (e.g., 1:0.3 or 1:0.2). In the 18th week, patients were encouraged to discontinue daily chemical bleaching and transition to maintenance therapy.

Outcome evaluation

The efficacy of the treatment was assessed in a blinded manner by two board-certified attending surgeons (MM and CT). They independently evaluated pre- and post-treatment photographs using the Melasma Area and Severity Index (MASI) scoring system [5], which ranges from 0 to 48 points, with lower scores indicating better treatment outcomes.

Statistical analysis

Statistical analysis was performed to assess differences in MASI scores before and after treatment. An unpaired t-test was used, with a significance threshold of p<0.05, due to its robustness against unequal variances and sample sizes among groups. All statistical analyses were conducted using GraphPad Prism 9.0 (GraphPad Software, San Diego, California, United States) and IBM SPSS Statistics for Windows, Version 28.0 (Released 2021; IBM Corp., Armonk, New York, United States).

## Results

This study included 27 patients (23 women and four men) with a mean age of 47.4 years. The target lesions were SL in 20 cases, melasma in 17, NCN in five, freckles in three, acquired dermal melanocytosis (ADM) in one, and SK in one. Eleven patients (40.7%) were suspected to have ACP. Our combination therapy was performed in six patients who agreed to our treatment strategy (Table 1).

**Table 1:** Patient list of the combination therapy MASI: Melasma Area and Severity Index; RA: retinoic acid; Fr: freckle; Me: melasma; NCN: nevocellular nevus; SK: seborrheic keratosis; SL: senile lentigo

No.	Sex	Age	Diagnosis	Treatment protocol	MASI score before the treatment	MASI score after the treatment	Adverse effects (except normal reaction to RA)	Completion status
1	F	60	Me+SL+NCN	The combination therapy (oral medications, chemical bleaching, and laser)	25.6	4.5	None	Completed
2	F	48	Me+SL+Fr		32.8	6.3	None	
3	F	48	Me+SL+NCN		22	1.8	Moderate erythema (RA reduced)	
4	F	66	Me+SL+SK		21.7	2.1	None	
5	F	45	Me+SL+NCN		7.6	0.6	None	
6	F	28	SL+Fr+NCN		4	1.2	None	
Mean		49.2			19	2.8		

**Figure 2:**
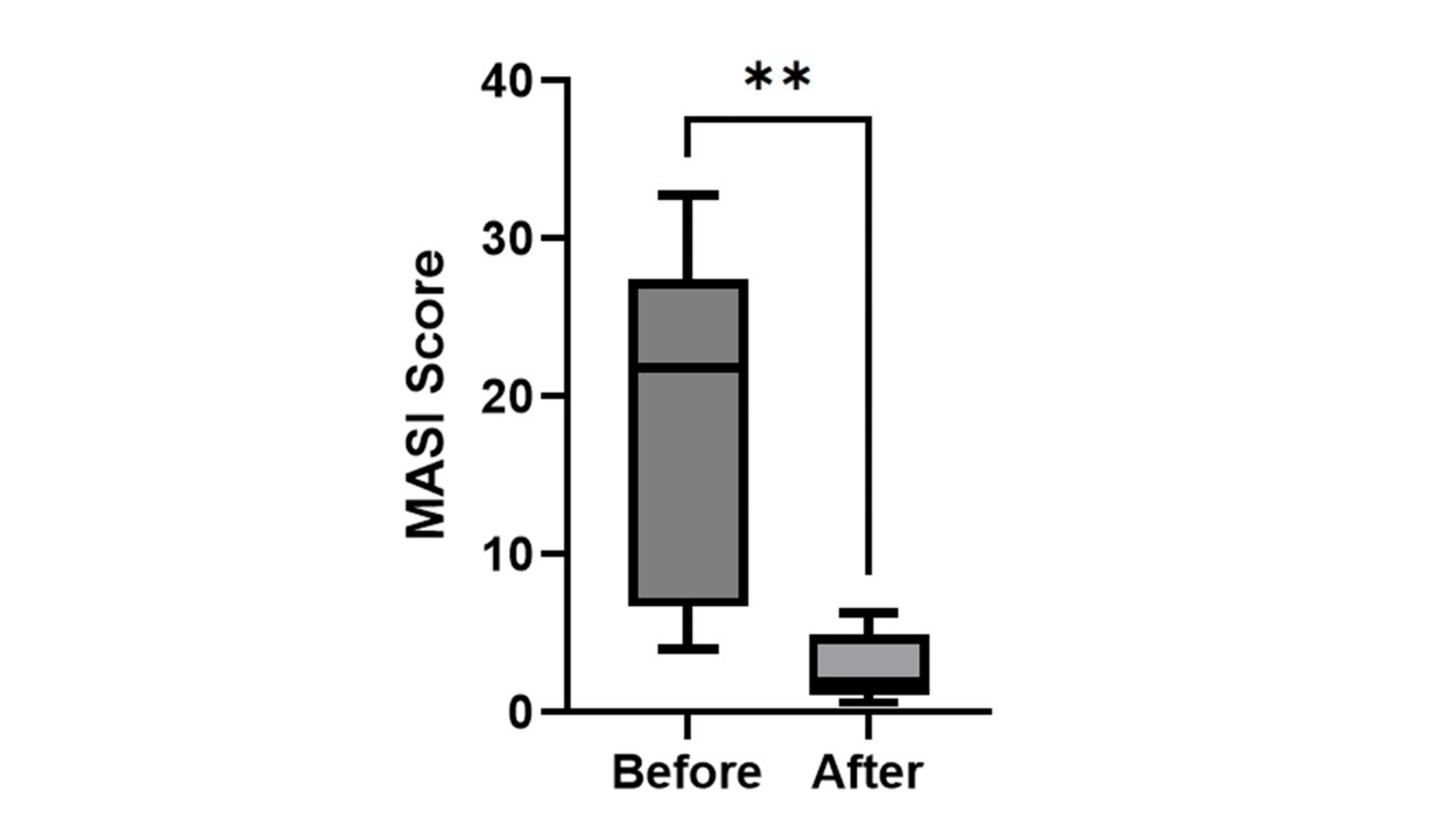
MASI score Statistical analysis revealed a significant difference in MASI scores before and after treatment (**p<0.01; unpaired t-test). MASI: Melasma Area and Severity Index

A moderate erythema was observed in one patient as an adverse reaction to RA, but by reducing the RA dosage by half, the erythema subsided to a mild level, which is considered normal. All six patients completed the treatment program without any dropouts. The mean MASI score of the six patients, as evaluated by the two attending doctors, was 19 before treatment and improved to 2.8 after treatment (p=0.0054; unpaired t-test) (Figure 2).

Representative case (no. 5 in Table 1)

**Figure 3:**
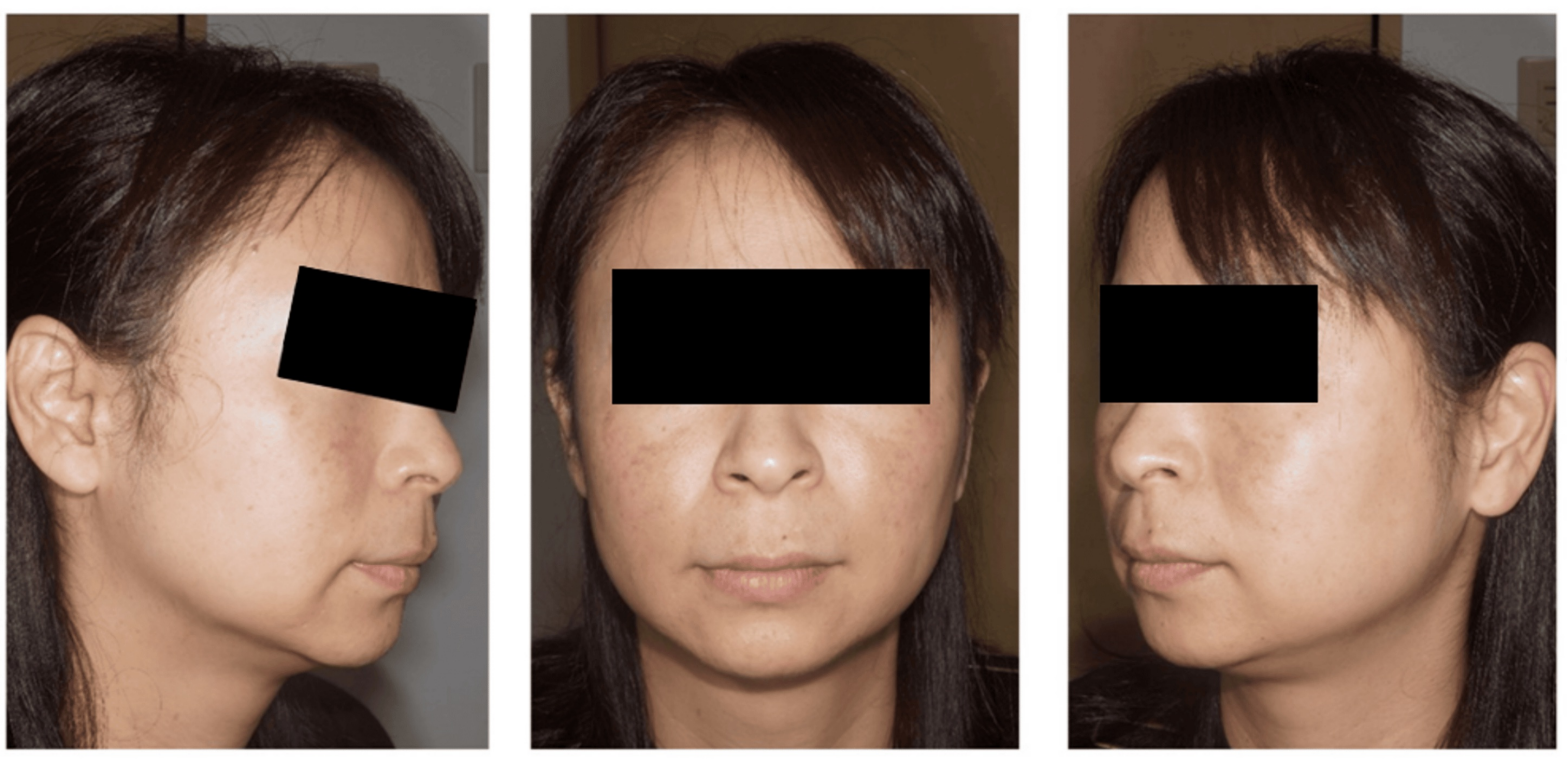
Case no. 5: before the treatment A 45-year-old woman with melasma and senile lentigo on her cheek. The average Melasma Area and Severity Index score evaluated by the two attending doctors was 7.6 before the treatment.

A 45-year-old woman presented with a chief complaint of pigmentation on her cheek. On examination, bilateral symmetrical sporadic pigmentation and non-symmetrical oval-shaped macules were observed on her cheeks (Figure 3). The initial clinical diagnosis by the resident was melasma and SL, which was later confirmed by two attending doctors. She underwent our combination therapy for ACP.

**Figure 4:**
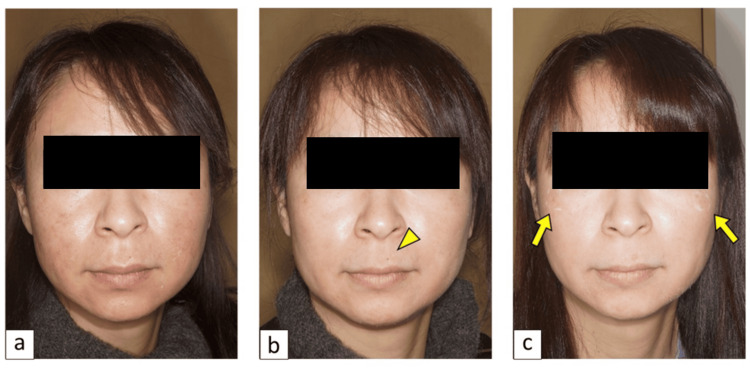
Case no. 5: clinical course of the protocol treatment (a) Mild exfoliation and erythema were observed during the first week after the initiation of chemical bleaching, within the normal range of adverse reactions to retinoic acid treatment. (b) A nevocellular nevus on the upper lip (arrowhead) underwent CO2 laser abrasion during the third week of chemical bleaching. The abraded area was covered with a hydrocolloid occlusive dressing for two weeks to protect it from daily chemical bleaching. (c) A senile lentigo on the cheek (arrows) was treated with Q-switched alexandrite laser irradiation during the sixth week of chemical bleaching, following two weeks of protection with tape covering. Notably, melasma was significantly alleviated by oral medication and chemical bleaching, allowing the remaining senile lentigo to be clearly distinguished.

Oral TA 1,000 mg/d, vitamin C 1,000 mg/d, and vitamin E 50 mg/d, concurrent with ZO®, were prescribed. She was instructed to follow our "1:0.5 policy," as described in the Methods section. Mild exfoliation and erythema were observed one week after the treatment (Figure 4a), indicating that daily chemical bleaching was performed correctly. The NCN on her upper lip underwent abrasion via CO2 laser in the third week (Figure 4b). Subsequently, SL was treated with Q-switched laser irradiation in the sixth week (Figure 4c). Daily chemical bleaching was discontinued in the 18th week, and she was transitioned to maintenance therapy. The patient reported that she was not disturbed by her facial pigmentation anymore, and she noted the rejuvenation effect of our treatment.

**Figure 5:**
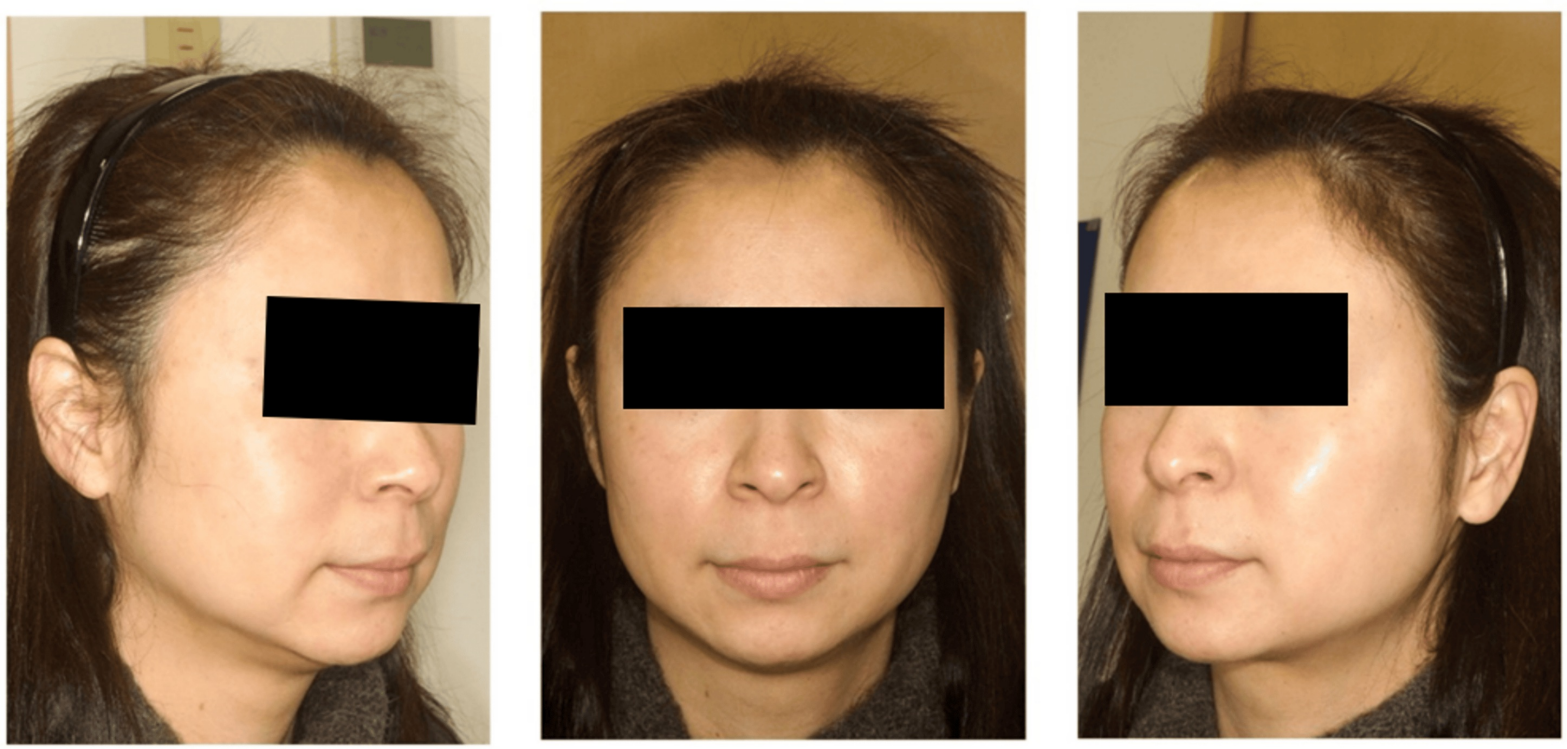
Case no. 5: six months after the treatment The combination therapy successfully eliminated the facial pigmentation and achieved favorable rejuvenation.

The outcome evaluation was conducted based on photographs taken six months after her initial visit (Figure 5). The average MASI score evaluated by the two attending surgeons was 7.6 before the treatment and improved to 0.6 after the treatment.

## Discussion

Patients visiting aesthetic dermatology clinics expect satisfactory treatment outcomes that justify the costs they incur. Meeting these expectations requires a high level of clinical expertise, which in turn necessitates thorough training. In particular, accurately diagnosing and managing ACP, commonly seen in middle-aged Asian women, is essential for providing appropriate treatment. However, most residents at the beginning of their careers lack the necessary skills to independently practice in an aesthetic dermatology clinic. As a result, plastic surgery residents typically focus on developing general clinical competencies first, which often limits their opportunities to gain hands-on experience in the field of aesthetic dermatology.

Our treatment protocol has been a breakthrough for residents in the current landscape, equipping them with a structured and comprehensive approach to effectively manage facial pigmentary disorders. By integrating oral medications, chemical bleaching, and laser therapy, this combination therapy addresses a wide range of common facial pigmentary conditions encountered in real clinical settings, offering a holistic solution for residents to gain confidence in their practice. A key feature of this protocol is the prioritization of oral medications and chemical bleaching for melasma treatment, which allows melasma to be controlled by the sixth week, reducing the risk of exacerbation due to the Q-switched alexandrite laser. Additionally, by ensuring consistent treatment quality, this protocol empowers residents with limited clinical experience to practice independently in aesthetic dermatology clinics.

The effectiveness of oral TA and vitamins for the treatment of melasma in Asian patients is widely recognized [6-8]. While caution is required regarding potential adverse effects of TA (e.g., gastric discomfort, thromboembolism) [9,10], oral medication remains an accessible treatment option for residents as it does not require specialized skills. Another common treatment option for melasma is chemical bleaching. In Japan, at-home chemical bleaching using HQ and RA ointments has been recognized as a conventional method [11]. However, this approach requires the preparation of ointments at each clinic, leading to challenges such as variability between facilities and the need for frequent patient visits. However, in 2006, Obagi launched ZO®, a commercialized HQ and RA ointment as part of an at-home facial rejuvenation program [12]. This development eliminated the variability between facilities and the need for frequent clinic visits, and it became widely adopted in Japan.

Another important feature of ZO® is that it allows patients to self-adjust the dose of RA in response to adverse reactions such as erythema, irritation, and exfoliation. We adopted a "1:0.5 policy" for the mixture: one push of Miramix® was mixed with half the amount of 0.1% RA. Patients were instructed to adjust the amount of 0.1% RA based on the severity of any adverse reactions they experienced. This flexibility helped alleviate discomfort caused by adverse reactions, ultimately contributing to the prevention of patient dropout from the treatment protocol.

Finally, combining chemical bleaching with laser therapy is expected to produce a synergistic effect on treatment outcomes. Chemical bleaching with HQ and RA reduces the overproduced melanin in the epidermis. The elimination of excess epidermal melanin allows laser light to penetrate deeper, enhancing the efficacy of Q-switched laser treatment for dermal lesions [13]. We basically perform Q-switched laser irradiation in the sixth week of the ZO® program. This timing coincides with the point when the therapeutic effects of chemical bleaching on melasma become evident, maximizing the efficacy of the laser treatment. In addition, the remaining 12 weeks of the ZO® program are expected to help reduce post-inflammatory pigmentation caused by the laser treatment [14], ultimately contributing to improved patient satisfaction.

It has been reported that the involvement of residents in aesthetic surgery is associated with longer operation times, which may lead to an increase in complication rates and associated costs [15]. Similarly, in aesthetic dermatology, factors that could potentially decrease patient satisfaction should be minimized as much as possible, which is why attending surgeons may hesitate to allow residents to participate directly. However, if high satisfaction outcomes can be guaranteed, attending surgeons can confidently delegate sole practice to residents. Our protocol treatment not only enables residents to practice independently and gain valuable clinical experience but also allows attending surgeons to save time that would otherwise be spent on teaching. This saved time can then be devoted to other academic activities (e.g., research), ultimately improving the overall productivity of the entire team.

One limitation of this study is its retrospective design, which may introduce bias in patient selection and treatment outcomes. Additionally, the sample size of 27 patients is relatively small, which may limit the generalizability of the results. The study also lacks a control group, making it difficult to compare the efficacy of the protocol against other treatment options. Another limitation is the reliance on a single treatment protocol, which may not account for the variability in response to treatment among different individuals. Finally, the follow-up period was not extended, so long-term results and potential recurrence of pigmentation could not be assessed.

In conclusion, our combination therapy is a safe and effective treatment program that produces satisfactory results, even when performed by a plastic surgery resident with limited clinical experience. It offers residents valuable hands-on experience in aesthetic dermatology with minimal risk of unfavorable outcomes. Furthermore, our protocol minimizes the time and effort required for supervision by attending surgeons, enabling more efficient clinical practice.

## Conclusions

Our protocol treatment offers an effective and safe approach for residents to gain hands-on experience in aesthetic dermatology with minimal risk of unfavorable outcomes. By combining oral medications, chemical bleaching, and laser therapy, the treatment addresses common pigmentary disorders while ensuring high patient satisfaction. Our protocol not only enables plastic surgery residents to practice independently but also minimizes the supervisory burden on attending surgeons. Ultimately, it enhances team productivity by allowing attending surgeons to dedicate more time to research and other academic endeavors.
